# [Retracted] Efficacy of thermosensitive chitosan/β-glycerophosphate hydrogel loaded with β-cyclodextrin-curcumin for the treatment of cutaneous wound infection in rats

**DOI:** 10.3892/etm.2025.12961

**Published:** 2025-09-02

**Authors:** Yong Zhao, Jia-Guo Liu, Wei-Min Chen, Ai-Xi Yu

Exp Ther Med 15:1304–1313, 2018; DOI: 10.3892/etm.2017.5552

Following the publication of this paper, it was drawn to the Editor's attention by a concerned reader that the β-actin western blots shown in Fig. 5D on p. 1310 were strikingly similar to data that appeared subsequently in the journal *Translational Cancer Research* in an article written by different authors at different research institutes, although the western bands in question in the latter paper were featured adjacent to an additional lane of data in the western blot. Moreover, certain of the histological data shown in Fig. 4C similarly appeared in a paper published subsequently in the journal *Drug Design Development and Therapy*, also written by different authors at different research institutes.

Given that the contentious data were published first in the above article, the authors were offered the opportunity to present their original (uncropped) data concerning the β-actin western blots in Fig. 5D*;* however, the authors weren't able to provide these original blots. Therefore, the Editor of *Experimental and Therapeutic Medicine* has decided that this paper should be retracted from the Journal on account of uncertainty concerning the original source of these published data. After contacting the authors, they accepted the decision to retract the paper. The Editor apologizes to the readership for any inconvenience caused.

